# A Resonator-Based Flexible Antenna for Non-Invasive Deep Brain Temperature Sensing with Microwave Radiometry †

**DOI:** 10.3390/s26051699

**Published:** 2026-03-08

**Authors:** Golap Kanti Dey, Mohammad Vaseem, Natalia K. Nikolova, Atif Shamim, Chih-Hung Chen

**Affiliations:** 1 Department of Electrical and Computer Engineering, McMaster University, Hamilton, ON L8S 4L8, Canada; deyg@mcmaster.ca (G.K.D.); chench@mcmaster.ca (C.-H.C.); 2 Department of Electrical and Computer Engineering, King Abdullah University of Science and Technology, Thuwal 23955-6900, Saudi Arabia; mohammad.vaseem@kaust.edu.sa (M.V.); atif.shamim@kaust.edu.sa (A.S.)

**Keywords:** flexible antenna, resonator, brain temperature, infant’s head, non-invasive, tissue layers, near-field directivity, microwave radiometry

## Abstract

We present a circular complementary split ring resonator (CCSRR) flexible antenna operating in the 1.4 GHz radio-astronomy quiet frequency band. The antenna is designed for microwave non-invasive brain temperature sensing of an infant’s head to aid in the therapeutic hypothermia treatment of hypoxic–ischemic encephalopathy (HIE) and traumatic brain injury (TBI). The proposed metamaterial-inspired antenna is designed on a flexible *Kapton* substrate with a biocompatible Polydimethylsiloxane (*PDMS*) protective superstrate layer. For brain temperature measurement, the flexible antenna is placed directly on the scalp to collect thermal noise power from the underlying tissue layers. The received thermal power is to be delivered to a sensitive microwave radiometer. The CCSRR antenna exhibits sharp frequency selectivity at 1.4 GHz with inherent filtering capability, strong field confinement, and excellent suppression of out-of-tissue (external) electromagnetic interference and thermal noise contributions. To closely match the realistic scenario, the CCSRR antenna, initially designed in a planar multi-layer configuration, is investigated in various bending configurations (cylindrical and spherical) with a curvature radius of 55 mm. The results indicate stable performance under bending. Good agreement between simulated and on-body measured results is observed in the desired frequency band.

## 1. Introduction

Biomedical sensing and imaging [1,2,3,4,5] are essential components of modern healthcare, aiding the diagnostics and the monitoring of various medical conditions. Common imaging modalities such as magnetic resonance imaging (MRI), computed tomography (CT), and ultrasound provide high-resolution deep-body images with varying, often complementary, capabilities in terms of tissue types, penetration, and sensitivity to tissue pathology. However, none of these modalities is suitable for prolonged monitoring due to exposure to ionizing radiation (CT), high cost (MRI), bulky equipment (MRI, CT), operator-specific images (ultrasound), and inability to provide patient comfort over a long time (CT, MRI, ultrasound).

On the other hand, the emerging microwave imaging and sensing biomedical technologies utilize the non-ionizing electromagnetic (EM) spectrum to characterize tissue properties, enabling the detection of anomalies such as tumors, brain injuries, abnormal blood glucose levels, and others [6,7,8,9,10]. These technologies offer significant potential for advancements in medical diagnostics due to the relatively low cost and small size of the equipment, as well as the advent of flexible substrates, antennas, and electronics.

Antenna technology plays a central role in radio frequency (RF), microwave, and millimeter-wave biomedical imaging and sensing. Recent advancements in material science have made biomedical antennas smaller, lightweight, and suitable for wearable and implantable devices. Human body antennas could be classified into on-body, off-body, and in-body categories [11], all of which are increasingly dependent on flexible electronics [12] for their effective operation. Flexible electronic systems offer unique capabilities in biomedical imaging and sensing, including very lightweight, biocompatibility, and excellent conformability to curved surfaces. Flexible antennas are being explored extensively to advance these capabilities while improving measurement accuracy, repeatability, and patient comfort [13,14,15,16].

A 16-element flexible antenna array has been proposed [17] for microwave-based breast cancer detection. This array includes single- and dual-polarized antennas (monopole and spiral) with a size of 20 mm × 20 mm on a 50 µm *Kapton* polyimide substrate to ensure flexibility, lightweight, and adaptability for wearable applications in the frequency band from 2 GHz to 4 GHz. A wideband flexible meander line antenna array is reported [18] for wearable head imaging systems. The antenna is fabricated using a 4 mm thick room-temperature-vulcanizing (RTV) silicone substrate and operates effectively over a wide frequency range of 0.45–3.6 GHz, where the reflection coefficient |*S*_11_| is less than −10 dB. A low-profile flexible MIMO antenna array (2 × 2) is reported in [19] to operate in the sub-6 GHz ISM band (5–6.6 GHz), targeting applications in wireless body area networks and biomedical telemetry devices. Overall, flexible antennas have demonstrated effectiveness and significant advantages in biomedical and healthcare applications by offering adaptability and patient comfort compared to rigid antennas.

In the past three decades, the deep-body temperature measurement has become a major focus of research [20,21,22,23] due to the lack of effective non-invasive solutions. The method relies on the measurement (typically in the low-GHz frequency range) of the thermal noise power emitted naturally by the human body, which serves as the foundation of the non-invasive temperature estimation. Microwave antennas are designed to capture this thermal noise power, which is proportional to the tissue absolute brightness temperature *T* as per the Nyquist–Johnson equation,

(1) P=kTΔf

where *P* is the noise power emitted by the tissue, *k* is the Boltzmann constant, *k* ≈ 1.38 × 10^−23^ J/K, and ∆*f* is the bandwidth of the receiver. Note that the antenna only collects the thermal power, which is then measured by the radiometer and is subsequently converted into a temperature reading through a series of system de-embedding steps, which take into account various system parameters such as: (i) the antenna reflection coefficient, physical temperature and efficiency, (ii) the efficiency of the interconnect between the antenna and the radiometer, (iii) the insertion loss, equivalent input noise temperature, noise figure, and gain variation of the components in the radiometer system, and (iv) rejection of electromagnetic interference (EMI) from the environment [24,25,26,27].

However, to our knowledge, all reported systems employ rigid antennas, which suffer from limitations in accommodating the complex body surfaces and the patient’s movement. The result is patient discomfort and poor skin contact, which may affect the measurement accuracy and the monitoring effectiveness [28]. In particular, when the rigid antenna structure does not realize uniform contact, the air gaps degrade the EM power coupling from the tissue, cause reflection mismatches, increase the susceptibility to EMI, and allow for out-of-body (external) noise power to interfere with the thermal radiation of the examined deep-body tissue. Ultimately, this degrades the measurement sensitivity, especially to thermal radiation from deeper body tissues. A detailed comparison between flexible and rigid antennas is provided in Table 1.

The continuous monitoring of the deep brain temperature is one of the most important applications of the biomedical temperature measurement systems. For therapeutic brain hypothermia treatment to be effective, it is imperative to have an accurate deep brain temperature reading. MRI, magnetic resonance spectroscopy, and infrared thermography [29,30,31,32,33] have been utilized to monitor non-invasively the temperature changes within the brain. However, MRI equipment is impractical in long-term monitoring, whereas infrared thermography suffers from limited assessment depth. Microwave radiometry (MWR) [24,25,34,35] offers an alternative non-invasive approach, which could effectively overcome these limitations. MWR has shown potential in several medical applications, including breast cancer detection [36,37,38,39], internal body temperature measurement [26,40,41,42,43], and monitoring brown fat metabolism [44].

**Figure 1:**
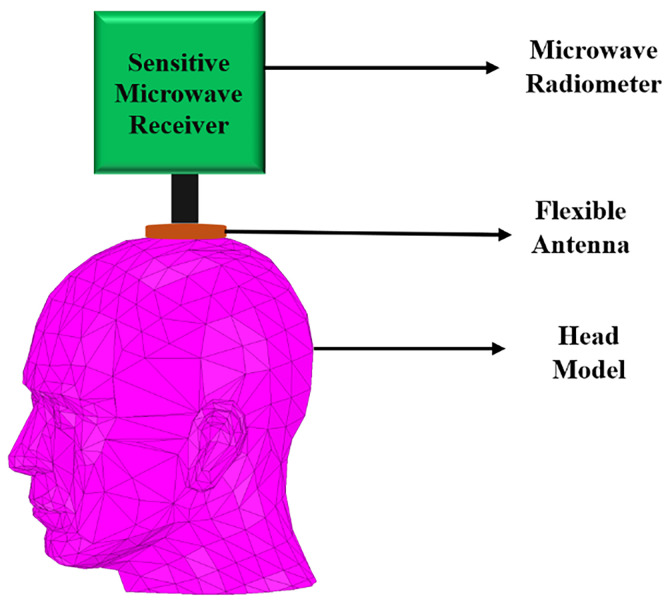
Illustration of a brain temperature monitoring system.

To our knowledge and to date, non-invasive solutions using flexible antennas have not been reported for the monitoring of deep brain temperature, including the case of the infant’s head during hypothermia treatment, which is the intended application here. To address this gap, a circular complementary split ring resonator (CCSRR) flexible antenna is proposed here to operate in direct contact with the scalp. However, the measurement of deep brain temperature is challenging due to the extremely low microwave radiation levels, which, according to (1), are about 4.3 × 10^−15^ W/MHz at a human body temperature of 37 °C. The CCSRR structure [45,46,47,48,49,50,51,52] is an excellent candidate for a narrowband unidirectional antenna with the ability to suppress out-of-band EMI while collecting maximum thermal vnoise power from the tissue. The power is then delivered to a sensitive noise power receiver, e.g., a microwave radiometer [53]; see Figure 1.

The novelty and main contributions of this work are summarized below:

1. Unlike previously reported rigid radiometric antenna solutions, this work establishes the design guidelines for a conformal flexible antenna, including frequency and bandwidth selection and the selection of suitable biocompatible flexible substrate, superstrate, and conductor materials.
2. A multi-layer EM infant head phantom is developed using literature-reported geometric and dielectric properties to enable the accurate evaluation of the antenna performance when operating in direct contact with the scalp.
3. A novel compact flexible CCSRR antenna is designed and optimized to achieve stable impedance matching under bending conditions, excellent near-field directivity (NFD), strong near-field confinement, and improved EM coupling in bending scenarios for deep brain temperature sensing.
4. Finally, the proposed thin flexible antenna is experimentally validated through measurements on both a cylindrical tissue-equivalent phantom and the human forehead, demonstrating excellent agreement with simulation results.

## 2. Design Procedure

**Figure 2:**
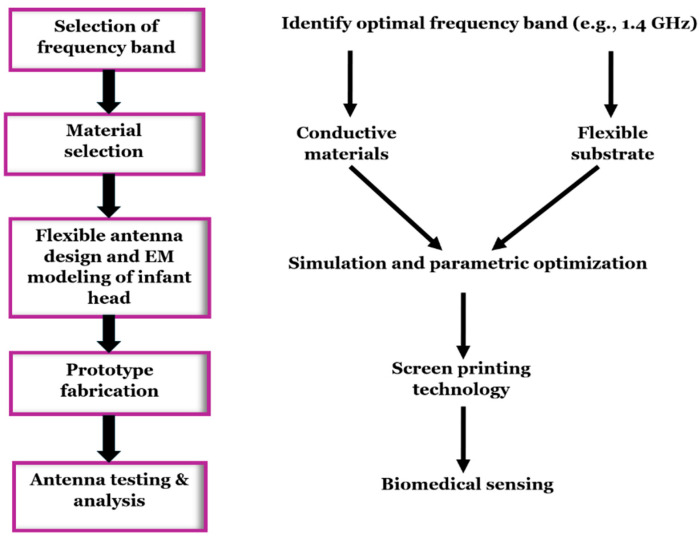
Design workflow for a flexible antenna.

The design procedure for flexible antennas (shown in Figure 2) begins with selecting the frequency band, followed by the selection of suitable flexible conductive and substrate materials based on their conductivity, permittivity, loss tangent, and biocompatibility.

The antenna is optimized through full-wave EM simulations, where the antenna is placed directly on the scalp of a realistic multi-layer numerical head phantom. The optimization is driven by performance metrics such as impedance match, near-field directivity, and near-field focusing with maximum penetration when the antenna operates in transmitting mode. Due to reciprocity, these metrics also describe the antenna performance in receiving mode, which is relevant in the passive noise power measurement systems. The design goal is to achieve impedance match better than −20 dB within the 1.4 GHz radio astronomy frequency band (1.400–1.427 GHz), an impedance bandwidth of at least 150 MHz at −10 dB level (in order to capture thermal noise power on the order of 10^−13^ W), and near-field directivity (as defined in [54,55]) that is better than 99% under various bending conditions to suppress effectively external EMI and thermal radiation. Also, the design aims to enhance the power density versus depth (in transmitting mode), which, by reciprocity, implies the ability to capture thermal power emitted from the deeper brain tissues.

### 2.1. Selection of Operating Frequency Band

The selection of the operating frequency is crucial for achieving a balance between spatial resolution, penetration depth, and avoiding EMI. After careful consideration of both the radio-astronomy frequency bands and the Industrial, Scientific, and Medical (ISM) frequency bands [56,57], the 1.4 GHz radio-astronomy frequency band has been selected. The reasons for this choice are several. First, the microwave radiation in the low-GHz spectrum has superior tissue penetration compared to the mm wave frequencies. It penetrates through the scalp, skull, cerebrospinal fluid (CSF), reaching well inside the brain tissues and enabling temperature sensing at a depth of several centimeters. At the same time, centimeter-scale spatial resolution inside the tissue is achievable.

Second, the radio-astronomy and space research frequency bands are safeguarded from the man-made interference; thus, they provide a “quiet” environment, free from industrial and communication EMI. This is important in brain temperature monitoring, where the thermal noise power emitted by the tissue is weak.

Lastly, the chosen 1.4 GHz frequency band is compatible with the in-house microwave radiometer prototype. We emphasize that although the bandwidth of the protected 1.4 GHz frequency band is limited to 27 MHz, the antenna is intentionally designed with a broader 10 dB impedance bandwidth of ~150 MHz to ensure sufficient thermal noise power for the microwave radiometer, which translates into better temperature sensitivity. Since a larger bandwidth may increase the system vulnerability to EMI, achieving near-field directivity (NFD) greater than 99% is also important.

### 2.2. Selection of Conductive Elements

Highly conductive materials like copper and silver nanoparticle inks are widely used for flexible antennas. To ensure stable performance under mechanical strain and deformation, various stretchable conductive materials such as silver nanowires (AGNWs), carbon nanotube-based polymers, and liquid metals have also been developed. Based on Table 2, silver is selected as a conductive material for the proposed flexible antenna design. Although copper has slightly higher bulk conductivity, silver has better thermal conductivity and resistance to oxidation, which is crucial in wearable sensors. On the other hand, compared with conductive polymers, nanocomposites, and liquid metals, silver ensures stable conductivity under bending and reliable adhesion to flexible substrates. Silver is also widely available and is suitable for low-cost flexible antenna fabrication processes.

### 2.3. Selection of Flexible Substrate

For biomedical applications, the flexible substrate material must be biocompatible, thermally compatible with the deposited conducting materials, and low-loss. Substrate materials used for flexible antennas are listed in Table 3. In this study, *Kapton* polyimide is used as the substrate, and a thin *PDMS* layer serves as the superstrate layer in the proposed flexible antenna. Altogether, the *Kapton* and *PDMS* combination achieves a balance among antenna electrical characteristics (such as impedance match and stable resonant behavior under bending), flexibility, and user comfort. In the proposed design, *Kapton* contributes to mechanical robustness and structural integrity, whereas *PDMS* enhances conformability and biocompatibility in addition to minimizing the mismatch due to the air gaps between the antenna and the skin surface.

### 2.4. EM Modeling of an Infant’s Head

To accurately simulate the antenna, the EM interaction with the head tissues must be properly represented through a realistic numerical head phantom. This is especially important when the antenna operates in direct contact with the scalp. The main tissue layers of the infant’s head model are the scalp, skull, CSF, and brain. The EM field behavior depends on the tissues’ dielectric properties, permittivity, and conductivity (or loss tangent), which vary with frequency. Also, the thickness of the scalp, skull, CSF, and brain tissues must properly represent the tissues in an infant’s head. Finally, the selection of the head diameter is important in the EM modeling of the antenna in the bending scenarios. The head size determines the curvature of the air–scalp interface, where reflections occur, impacting the power distribution within the cranial structure. In this work, a head radius of 55 mm has been selected, which corresponds to the average head circumference of ~35 cm of a newborn [67,68].

**Figure 3:**
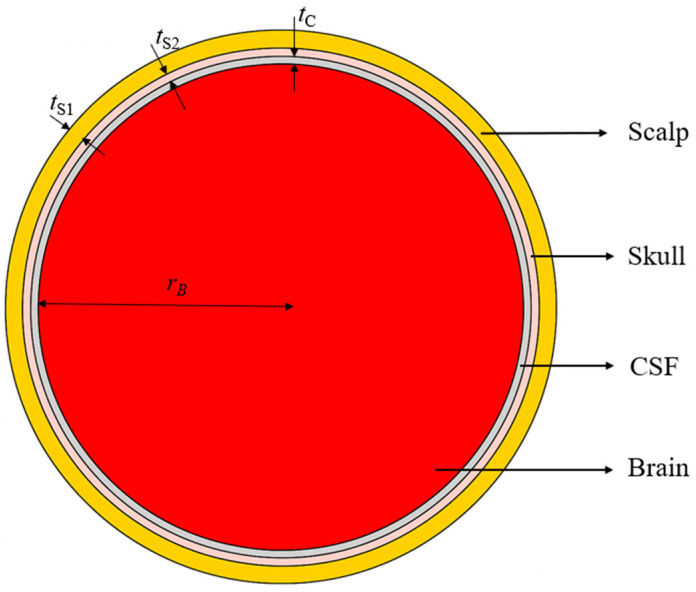
Cross-sectional view of the spherical infant’s head model. Dimensions in mm: *t*_S1_ = 3.5, *t*_S2_ = 1.5, *t*_C_ = 1.5, *r*_B_ = 55.

In this study, a four-layered head model is developed, consisting of the scalp, skull, CSF, and brain tissues. The tissues’ permittivity and conductivity values are calculated at the center frequency 1.413 GHz of the 1.4 GHz radio-astronomy band from established databases [69,70], where these properties are available as functions of frequency. The layer thicknesses are chosen from reported anatomical measurements [71,72,73,74]. The used tissue EM properties and thicknesses are summarized in Table 4. Figure 3 shows the cross-sectional view of the spherical four-layer infant’s head model. The scalp forms the outer layer of the head model, followed by the skull, CSF, and brain tissue.

### 2.5. Flexible Antenna Geometry and Multi-Layer Stack-Up

Although antenna miniaturization can be achieved by employing high-permittivity substrates, this approach is limited by the lack of high-permittivity flexible and biocompatible materials. To overcome this limitation and to maintain high sensitivity for near-field sensing, a metamaterial-inspired CCSRR antenna is proposed for operation on the four-layer tissue phantom. A full ground plane with a radius of 30 mm is used on the back side to ensure shielding from external electromagnetic interference (EMI) and improved unidirectional radiation. The designed CCSRR structure features a high Q-factor for narrowband operation centered in the 1.4 GHz radio-astronomy band. CCSRR structures behave effectively as resonant LC circuits [46]. The circular slot geometry increases the effective current path, contributing to inductive behavior, while the split-ring gaps introduce capacitive coupling between adjacent sections. The interaction of these equivalent inductive and capacitive components establishes a localized resonant mode, resulting in high-Q operation. This resonant mechanism enables compact size and enhances the field confinement, thus improving the suitability for radiometric sensing. Compared to a conventional patch, slot, or spiral antennas, CCSRR structures provide better field confinement and a smaller physical footprint for a given frequency of operation. Furthermore, the circular geometry ensures rotational symmetry and stable resonance under bending, which is important for flexible and conformal deployment on curved body surfaces, such as the infant’s head.

**Figure 4:**
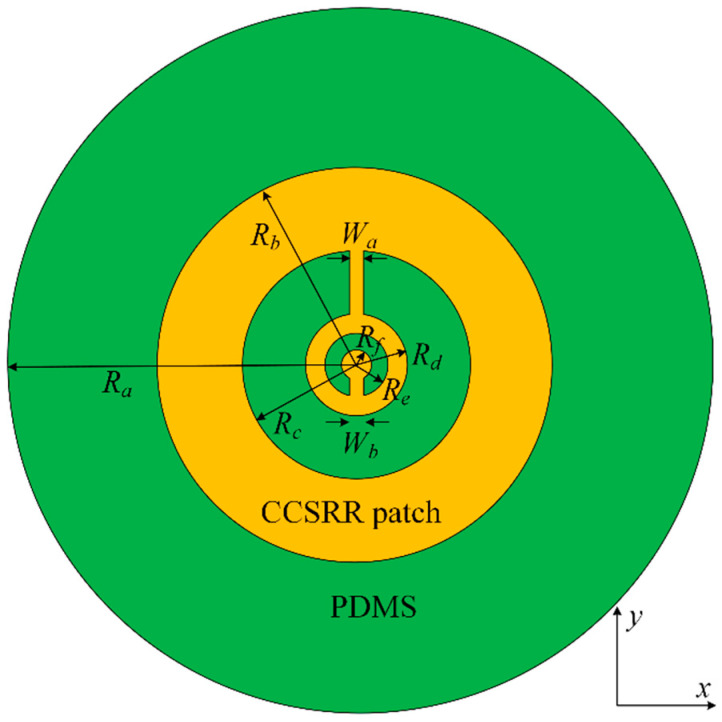
Geometry of the CCSRR radiating structure facing the tissue medium. The dimensions (all in mm) are: *R_a_* = 30; *R_b_* = 14; *R_C_* = 8.6; *R_d_* = 3.55; *R_e_* = 2.55; *R_f_* = 1.3; *W_a_* = 1; *W_b_* = 1.

**Figure 5:**
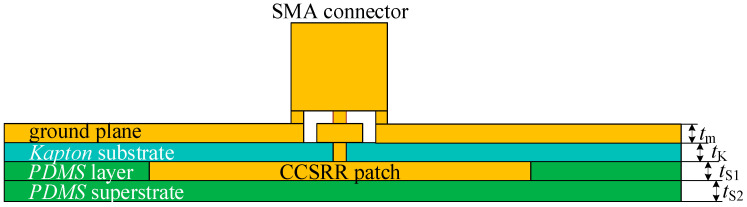
Stackup of the flexible multi-layer structure of the CCSRR flexible antenna. The dimensions (all in mm) are: *t*_m_ = 0.035; *t*_K_ = 0.125; *t*_S1_ = 0.035; *t*_S2_ = 0.015.

The antenna consists of two metallizations and two dielectric layers (substrate and superstrate). The metallization layer (silver) of the radiating CCSRR patch (facing the tissue) is shown in Figure 4. The 35 µm thick CCSRR patch lies on the 125 µm thick flexible *Kapton* substrate with a relative permittivity of 3.5 and a loss tangent of 0.007. A *PDMS* layer of 35 µm thickness is added around it to remove the air gap between the *Kapton* substrate and the *PDMS* superstrate; this is also indicated in the stackup in Figure 5. The thin biocompatible *PDMS* superstrate layer (relative permittivity of 2.55 and loss tangent of 0.01) is 15 µm thick, and it works as an insulation between the antenna and the scalp. It protects the metallic patch from oxidation and corrosion, ensuring durability and consistent performance when in direct contact with the skin. The superstrate thickness is tuned for a smooth impedance transition between the antenna and the tissue medium, thus improving the power coupling. The full ground plane on the backside of the antenna structure, where the coaxial connector is placed, is also realized using silver. The ground plane is significantly larger than the CCSRR patch, which ensures proper shielding from external EMI and unidirectional reception from the tissue. The optimized dimensions of the CCSRR structure for resonance at 1.413 GHz are detailed in Figure 4 and its caption. The cross-sectional view of the multi-layer stack in Figure 5 is also accompanied by the respective thickness values in the caption. The overall antenna performance is mainly controlled by the resonator radius, slot width, gap spacing, substrate thickness, and ground plane size, which determine the effective inductance–capacitance behavior and resonance characteristics. These parameters influence the operating frequency, resonance selectivity, and field confinement.

## 3. Results

**Figure 6:**
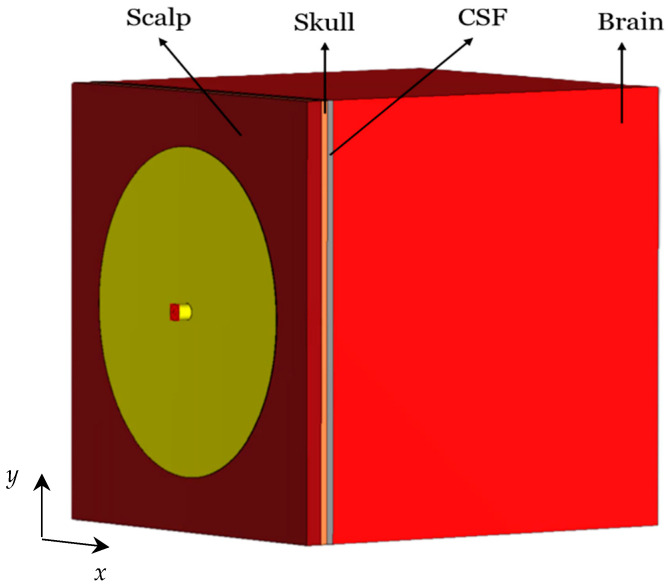
Flexible CCSRR antenna loaded with a four-layered planar tissue phantom.

To evaluate the performance of the designed CCSRR-based flexible antenna, initially, it is simulated while in direct contact with a planar four-layer numerical head phantom, as shown in Figure 6. The tissue layers are assigned the dielectric parameters listed in Table 4.

**Figure 7:**
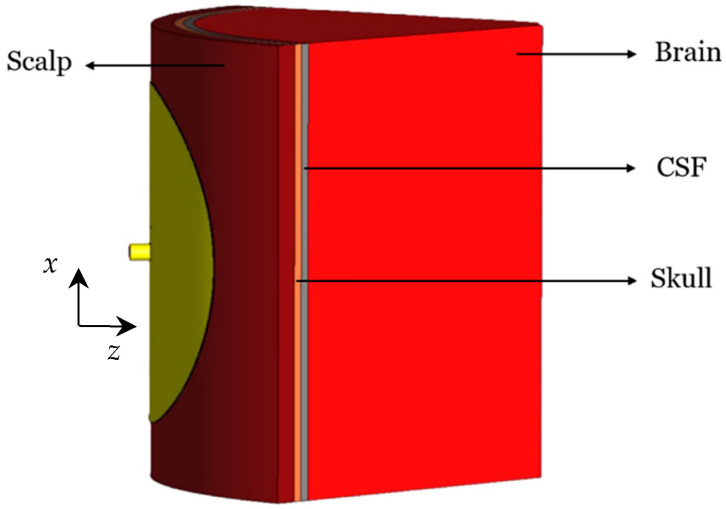
Cylindrically bent (*x*-axis) CCSRR antenna with a 55 mm curvature radius.

**Figure 8:**
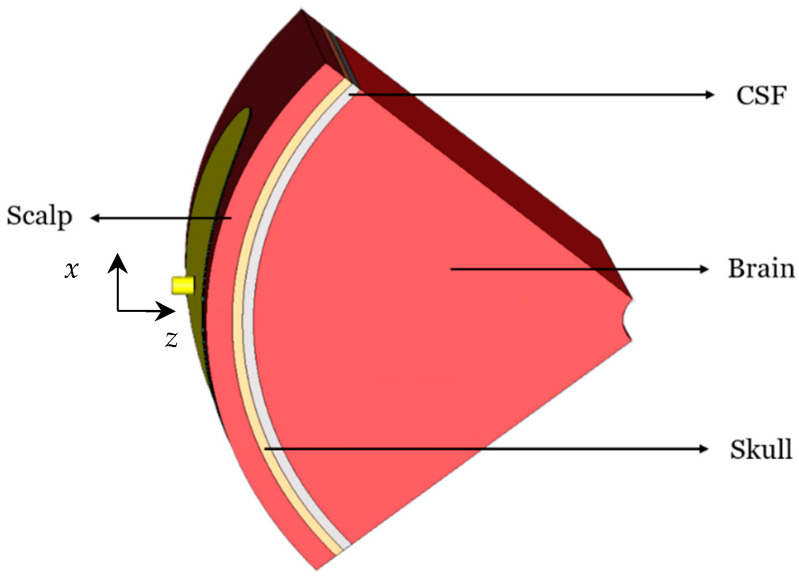
Cylindrically bent (*y*-axis) CCSRR antenna with a 55 mm curvature radius.

**Figure 9:**
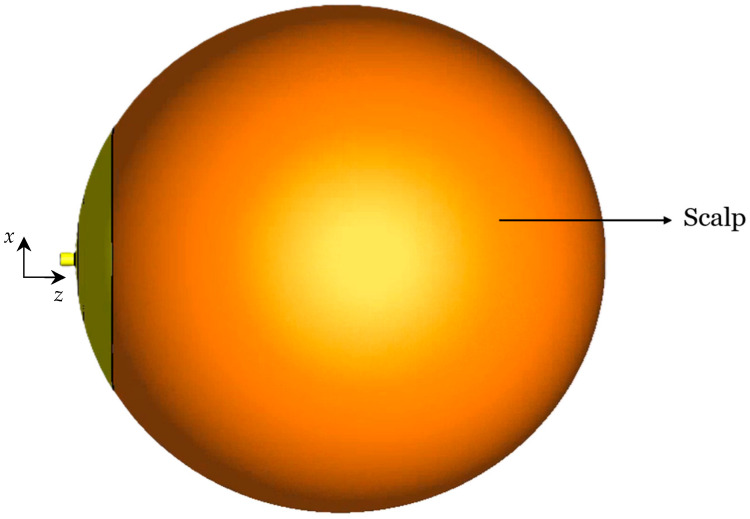
Spherically bent CCSRR antenna with a 55 mm curvature radius.

Since in practice the antenna must also conform to the curved surface of an infant’s head, the bending effects are also investigated as they influence the resonant frequency, the impedance bandwidth, the NFD, and the near-field distribution of the antenna. Four bending scenarios are investigated, where the curvature radius is always 55 mm, matching the radius of a newborn’s head. The first investigated cylindrical bending geometry (around the *x*-axis) is shown in Figure 7. The second cylindrical bending (around the *y*-axis) is shown in Figure 8. Finally, the spherical bending scenario is shown in Figure 9.

### 3.1. S-Parameter Responses

**Figure 10:**
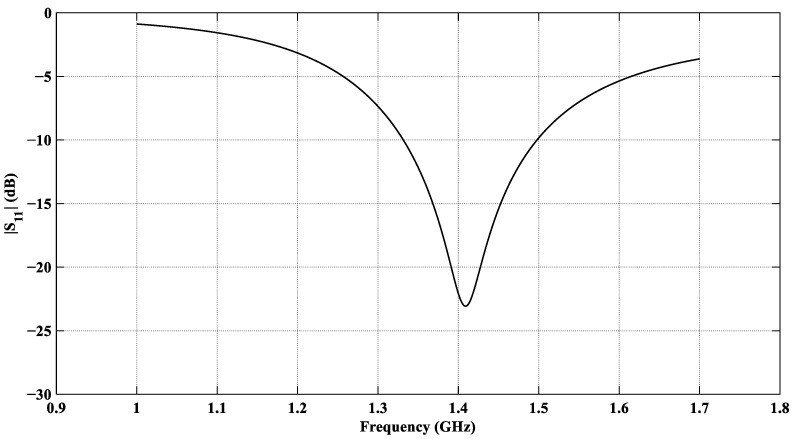
|*S*_11_| response of the designed CCSRR-based antenna with the planar tissue model.

The EM performance of the designed antenna is evaluated using the Finite Element Method (FEM) simulator in CST Microwave Studio [75]. The antenna is optimized for a minimum reflection coefficient at *f*_0_ = 1.413 GHz. The reflection coefficient |*S*_11_| of the CCSRR antenna loaded with the planar tissue model is shown in Figure 10. It shows a satisfactory value of –23 dB at *f*_0_ = 1.413 GHz.

**Figure 11:**
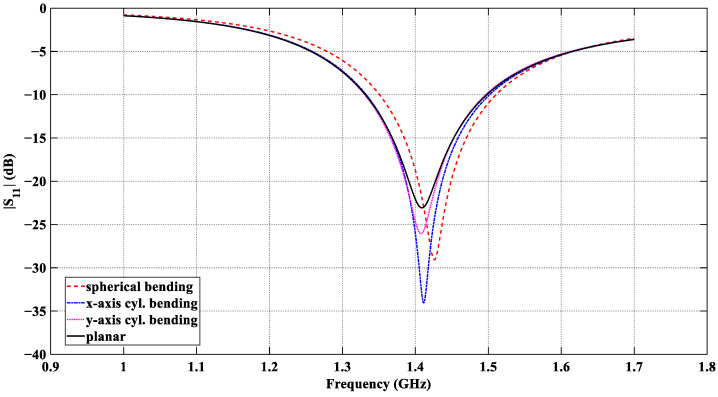
|*S*_11_| response of the designed CCSRR antenna with the four tissue models: planar, *x*-axis cylindrical bending, *y*-axis cylindrical bending, and spherical bending, all with a curvature radius of 55 mm.

The results in Figure 11 confirm that effective power coupling from the tissue medium to the antenna port is realized in all four scenarios. The impedance-match bandwidth and the center frequencies obtained with all bending configurations are summarized in Table 5. In all cases, the resonant frequency of the flexible antenna is near 1.413 GHz; its reflection coefficient is consistently below −20 dB in the entire 1.4 GHz radio-astronomy band, and the impedance bandwidth remains in the range from ~160 MHz to ~170 MHz (at the −10 dB level).

### 3.2. Near-Field Directivity

**Figure 12:**
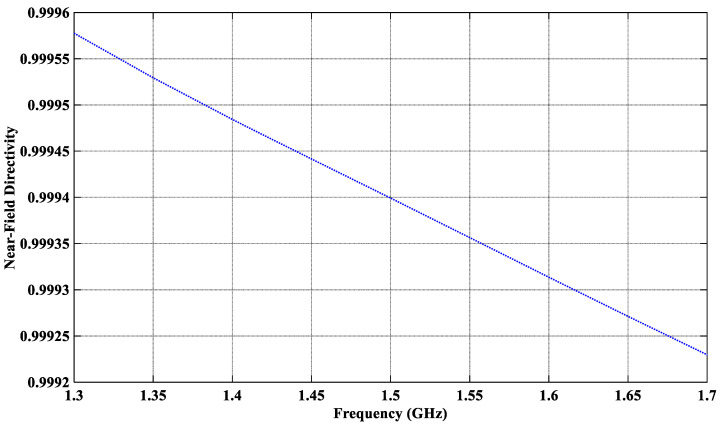
Near-field directivity of the proposed CCSRR antenna in the planar scenario.

The effectiveness of the antenna to suppress external EMI is assessed through its near-field directivity (NFD), *D*_nf_. In a transmitting (Tx) mode, the NFD is the ratio of the power coupled into the tissue $P_{\mathrm{rad}}^{\mathrm{tsu}}$ and the total radiated power $P_{\mathrm{rad}}$ in all directions, i.e., $D_{\mathrm{nf}}=$$P_{\mathrm{rad}}^{\mathrm{tsu}}/P_{\mathrm{rad}}$ [54,55]. By reciprocity, in a receiving mode, *D*_nf_ is the ratio of the power received from the tissue and the total received power. The power calculation uses a rectangular box, which encloses the whole antenna, including the coaxial connector. The front face of the box, where $P_{\mathrm{rad}}^{\mathrm{tsu}}$ is computed, overlaps the interface between the superstrate layer and the scalp. The power radiated through each face is calculated by surface integration of the real part of the normal component of the Poynting vector. The proposed antenna features an NFD of over 99.94% at the center frequency (see Figure 12), i.e., only 0.06% of the total received power is attributed to external (out-of-tissue) sources, which are the potential EMI sources. The NFD remains relatively insensitive to bending.

### 3.3. Power Flow Distribution of the Proposed CCSRR Antenna in the Planar Model

**Figure 13:**
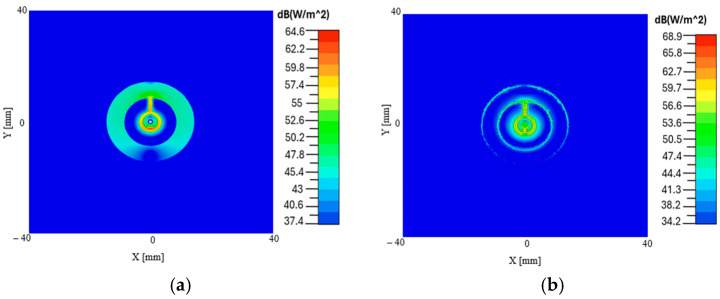

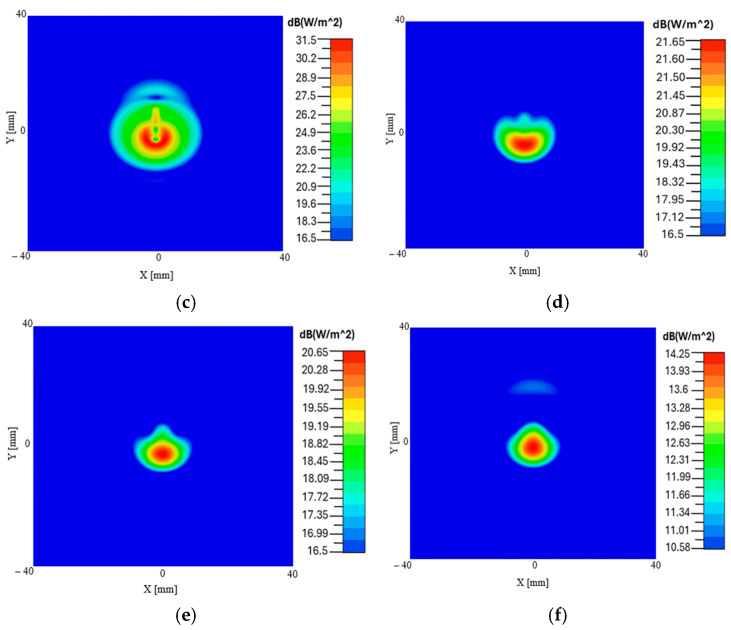
Distribution of the Poynting vector magnitude (in dB) in the planar configuration: (**a**) in the middle of the *Kapton* substrate, (**b**) in the middle of the *PDMS* superstrate, (**c**) in the middle of the scalp tissue layer, (**d**) in the middle of the CSF layer, (**e**) at the top of the brain tissue layer, (**f**) 5 mm inside the brain.

Figure 13 illustrates the lateral near-field distribution of the proposed CCSRR-based antenna at various depths as it propagates through the planar multi-layer structure. Note that in the simulations, the antenna operates in a Tx mode. The plots show how the antenna couples power into different layers starting from the substrate and ending at 5 mm inside the brain tissue layer. By reciprocity, the distributions also describe the sensitivity profile of the radiometric system. Specifically, Figure 13 shows the Poynting vector magnitude distribution in the lateral (along *x* and *y*) planes at different depths. We observe that the field remains highly focused as it propagates into the deep tissue regions, particularly within the brain layer. This indicates that the designed antenna can capture measurable EM power from the deeper intracranial regions, which is the prerequisite for localized radiometric temperature sensing.

### 3.4. E-Field Distribution of the Proposed CCSRR Antenna in the Planar Model

**Figure 14:**
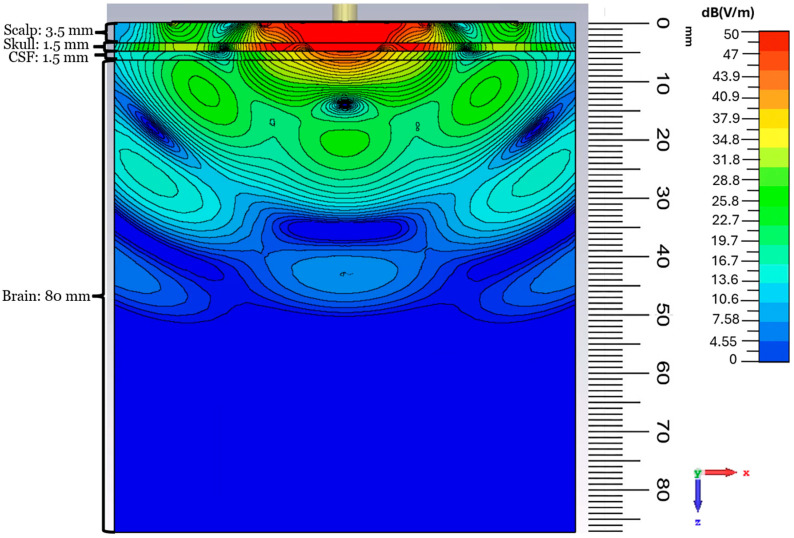
*E*-field magnitude distribution (in dB) in the case of the planar model in the *xz* plane at 1.413 GHz.

The *E*-field depth distribution in the planar configuration is shown in Figure 14. A concentrated high-field region exists in the scalp, skull, and CSF layers, beneath the antenna, with good near-field focusing. As the field propagates into the brain tissue, it decays nearly exponentially with depth, which is expected as the medium is very lossy. The observed field distribution features a focused profile with limited lateral spread. By applying the reciprocity theorem and using the simulated depth distribution, we can estimate that the designed flexible antenna enables capturing thermal radiation from a depth of up to ~45 mm. The simulated field strength drops from ~50 dB(V/m) at the scalp to ~10 dB(V/m) at a depth of 45 mm, which corresponds to an attenuation of ~40 dB. At a temperature of 37 °C, and with a bandwidth (BW) of ~160 MHz, the thermal power emitted by the brain tissue is on the order of 10^−13^ W, as per Equation (1). With the estimated ~40 dB attenuation due to a signal path of ~45 mm through the tissue, the power level reaching the radiometer input is at a level of ~10^−17^ W. This power level is close to the temperature sensitivity of advanced radiometers (less than 0.1 °K) [76,77] and is consistent with the detection limit of modern microwave radiometers [41,43,78], when the integration time is on the order of seconds. This confirms the feasibility of practical temperature sensing when the proposed flexible antenna is coupled with a sensitive microwave radiometer.

### 3.5. E-Field Distribution Under Cylindrical Bending

**Figure 15:**
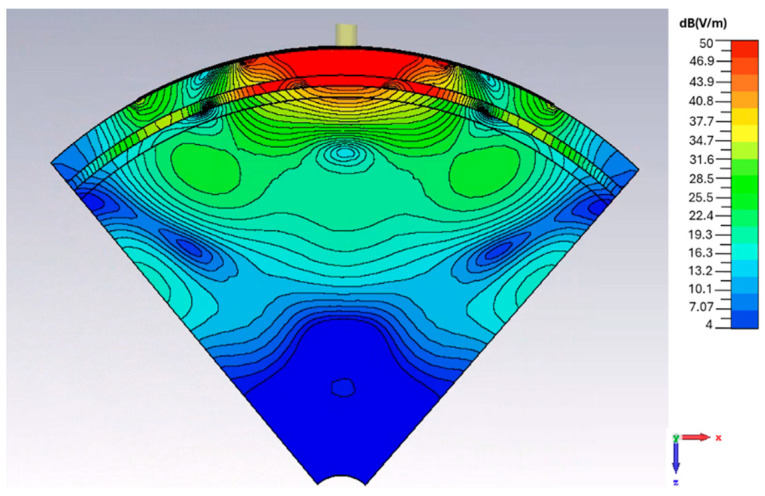
*E*-field magnitude distribution (in dB) in the case of cylindrical *y*-axis bending in the *xz* plane at 1.413 GHz.

To observe the effect of bending on the field depth distribution, the *E*-field magnitude distributions for the *y*-axis cylindrical bending are reported in Figure 15. The *E*-field remains strongly concentrated beneath the antenna, and, similarly to the planar case, propagates predominantly along the normal direction into the tissue layers. The depth profile inside the brain shows the same near-exponential attenuation with depth, which confirms that the bending does not degrade the penetration performance. For example, from the antenna center to a depth of ~30 mm, the field strength remains at the ~29 dB(V/m) level in both the planar and the cylindrical cases, which is about a 20 dB drop in field strength from the top of the scalp.

### 3.6. E-Field Distribution Under Spherical Bending

**Figure 16:**
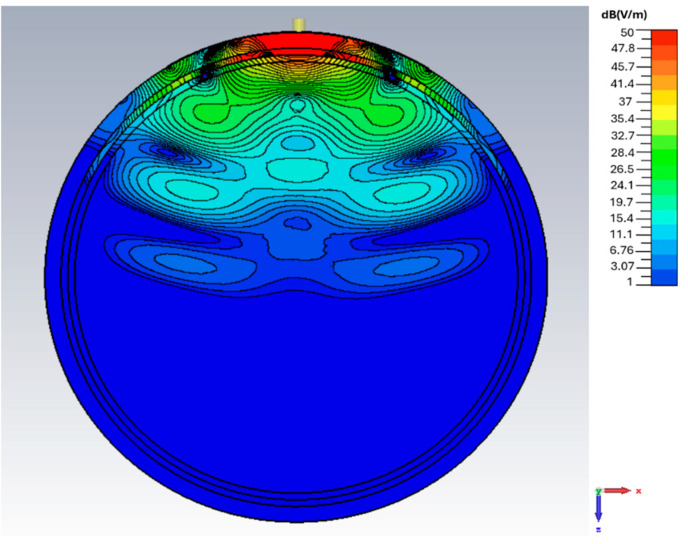
*E*-field magnitude distribution (in dB) in the case of spherical bending in the *xz* plane at 1.413 GHz.

The depth distribution of the spherical bending scenario with a curvature radius of 55 mm is shown in Figure 16. A strong and localized field region forms in the scalp, skull, and CSF layers. At a depth of ~30 mm, the field strength remains at ~29 dB(V/m), similar to the planar and cylindrical bending scenarios under identical tissue loading conditions. At the depths approaching ~45 mm within the brain, the field strength drops by ~45 dB(V/m) compared to that at the interface between the antenna and the scalp.

### 3.7. Comparative Analysis of Power Density vs. Depth

The flexible antenna design aims to capture thermal radiation from the deepest regions in the brain. The average power density dependence on the depth (when the antenna is in Tx mode), as measured from the antenna–skin interface, provides a quantitative estimation of this capability. The power flux density is averaged over an area at any depth position rather than taking a single point along the *z*-axis. A power flow monitor is defined in the simulation environment at 1.413 GHz to extract the average power flux density on multiple *z*-planes at different depths, where each plane covers a 60 mm × 60 mm square plane beneath the antenna. In this model, the brain tissue starts at 6.5 mm from the skin surface; thus, depths from 6.5 mm to about 45 mm define the region of interest.

**Figure 17:**
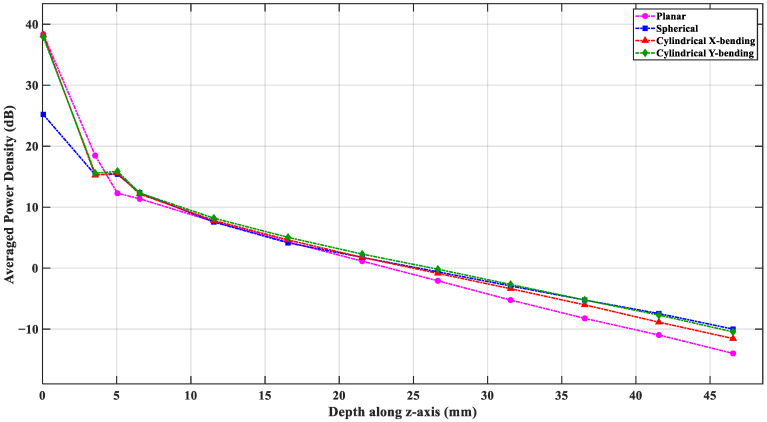
Comparative analysis of averaged power density vs. depth in the four investigated cases.

Figure 17 shows the averaged power density *vs*. depth in the planar and the three bending scenarios. Overall, all four configurations show a near-exponential power decay as the depth increases. The planar structure has the lowest power values beyond depths of 20 mm, whereas the spherical structure maintains the highest power levels in the deepest parts of the brain. At 40 mm depth, the cylindrical and spherical bending configurations show about ~3 dB more power density compared to the planar scenario. This improvement continues toward larger depths. By reciprocity, this improvement indicates that the curvature of the bending focuses the antenna sensing region and enhances the EM power coupling from the deep tissue layers. This also implies better sensitivity to thermal emission from the core brain regions when the antenna is bent around the head.

### 3.8. Forehead-Mounted S-Parameter Charterization

**Figure 18:**
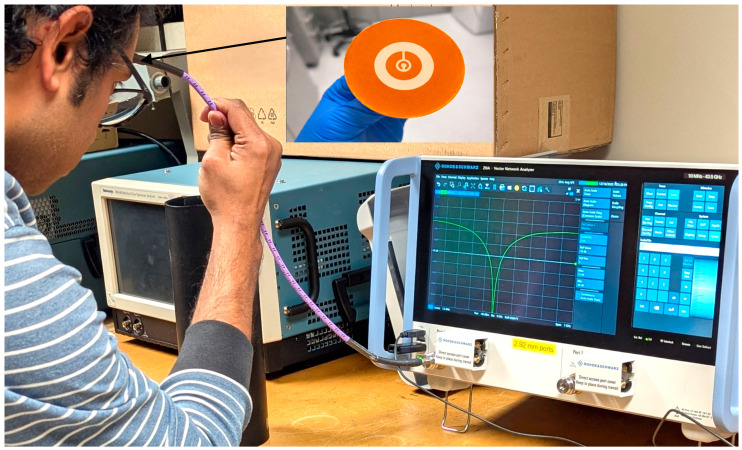
Measurement setup on a volunteer’s forehead with the fabricated flexible CCSRR antenna.

The proposed CCSRR-based flexible antenna is fabricated using low-cost screen-printing technology. The designs are created using a laser-cut PI tape-based mask, as described in [79]. The mask is attached to a blank mesh screen (120 mesh count), and screen printing is performed using a professional printing system (AUREL screen printer 900PA, AUREL S.p.A., Via Foro dei Tigli, 4, 47015 Modigliana FC, Italy). The CCSRR and ground plane are screen-printed at a printing speed of 300 mm s^−1^ using a commercial silver paste (DM-SIP-3072S, Dycotech, Dycotec Materials Ltd Unit 6, Srainer Road Porte Marsh Industrial Estate Calne SN11 9PX Wiltshire, UK) and subsequently annealed at 120 °C for 1 h to achieve a conductivity of 1.2 × 10^7^ Sm^−1^. The PDMS-based ink is formulated as described in [80] and screen-printed at a printing speed of 100 mm s^−1^ onto the designated area, followed by drying at 100 °C for 30 min in a vacuum oven. Finally, via filling is performed, and the SMA connector is mounted using a conductive silver epoxy (DM-SAS-10030-SYP, Dycotec Materials Ltd Unit 6, Srainer Road Porte Marsh Industrial Estate Calne, SN11 9PX Wiltshire, UK) for measurement purposes. Following the antenna fabrication, the measurement is conducted directly on an adult human forehead to evaluate its impedance match under realistic on-body conditions; see Figure 18. Although the intended application targets infant brain temperature sensing, the differences in the head geometry and the dielectric properties between adult and infant heads mainly affect the penetration depth rather than the fundamental resonance behavior of the antenna and, therefore, its impedance match. Thus, the adult forehead measurement provides a valid preliminary validation of the antenna impedance match and its conformal performance. The S_11_ parameter measurements are performed using a vector network analyzer (VNA) [81]. Single-port VNA calibration is performed using the electronic calibration module.

### 3.9. Cylindrical Bending Characterization and Brain Tissue Equivalent Phantom Preparation

**Figure 19:**
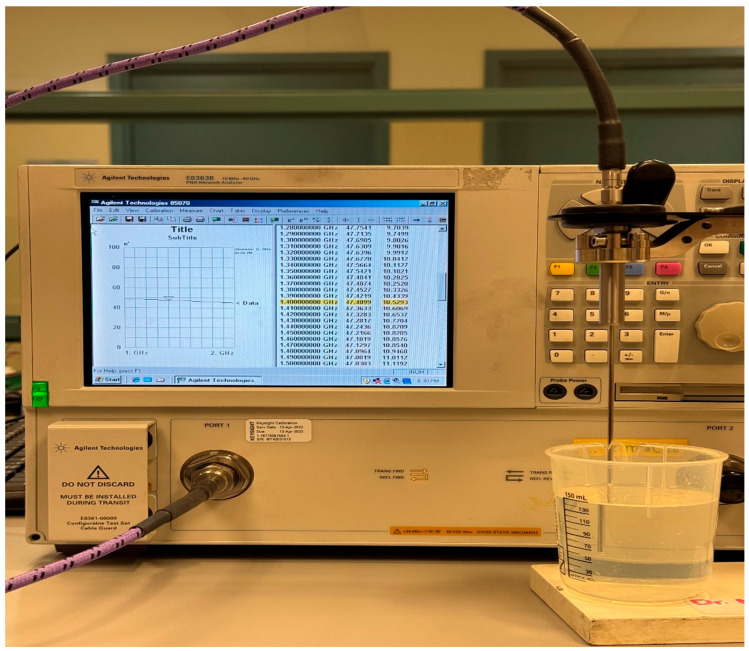
Measurement setup for dielectric characterization of the prepared brain tissue-equivalent liquid using the Keysight N1501A dielectric probe kit connected to a VNA [84].

**Figure 20:**
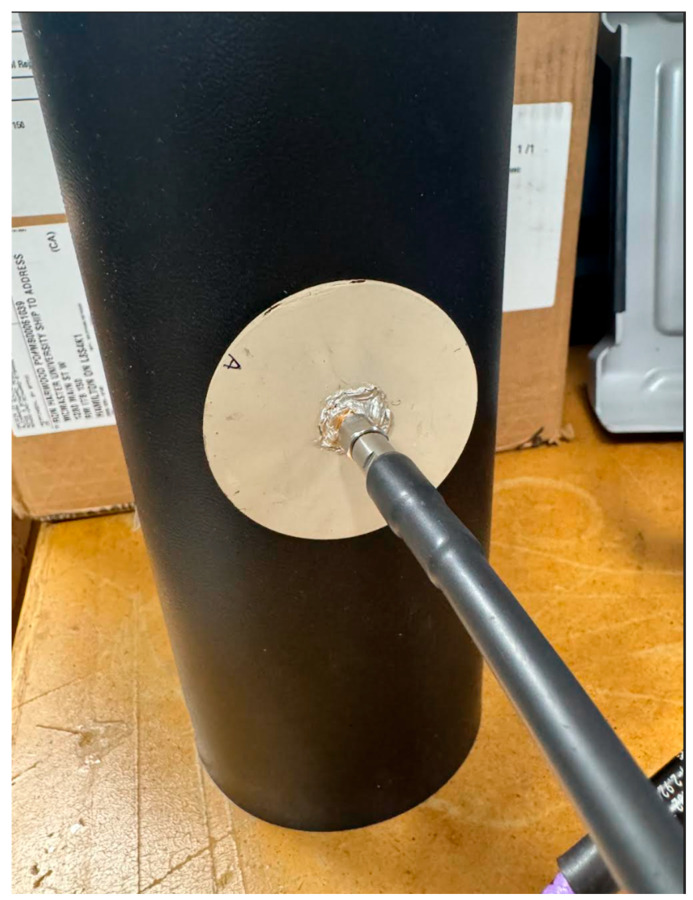
Conformal bending measurement of the flexible CCSRR-based antenna on the prepared cylindrical phantom with a 55 mm radius during the *S*_11_ measurement.

The antenna performance under cylindrical bending is evaluated using an in-house cylindrical phantom with a 55 mm radius. The phantom is prepared using commercially available microwave absorber material (JCS-9) [82] with a 2 mm thickness wrapped around a cylindrical plastic container with an inner radius of 52 mm filled with a brain tissue-equivalent liquid. The wall thickness of the container is 1 mm. The liquid is prepared using a mixture of isopropyl alcohol and distilled water to achieve the target relative permittivity reported in Table 4. A linear volume fraction mixing model is applied, where the effective complex permittivity of the liquid $\varepsilon_{L}$ is expressed as

(2) $$\varepsilon_{L}=(1-\varphi )\varepsilon_{A}+\varphi \varepsilon_{W}$$

where φ is the volume fraction of water and $\varepsilon_{A}$ and $\varepsilon_{W}$ are the relative permittivities of isopropyl alcohol and water, respectively. The measured complex permittivity values at 1.4 GHz for isopropyl alcohol and water, measured with Keysight N1501A dielectric probe [83], are 26.83 − *i*10.31 and 75. 12 − *i*5.85, respectively. Using $\varepsilon_{A}$= 27 and $\varepsilon_{W}$= 75, and the targeted permittivity $\varepsilon_{L}$ = 47, the distilled water fraction is calculated as φ = 0.417. Based on this result, a total volume of 150 mL is prepared by mixing 85 mL of isopropyl alcohol with 65 mL of distilled water. The same volumetric ratio is maintained while scaling the total mixture volume to completely fill the cylindrical container, ensuring consistent dielectric properties throughout the phantom during measurement. The dielectric properties of the prepared liquid phantom are measured and verified using the dielectric probe kit, as shown in Figure 19. The measured complex permittivity of the prepared liquid phantom is 47.41 − *i*10.52 at 1.4 GHz. The flexible antenna is then conformally bent along the prepared cylindrical phantom; see Figure 20, and the *S*_11_ parameter measurements are carried out to evaluate impedance stability under combined cylindrical bending and on-body conditions.

### 3.10. Experimental S-Parameter Characterization

**Figure 21:**
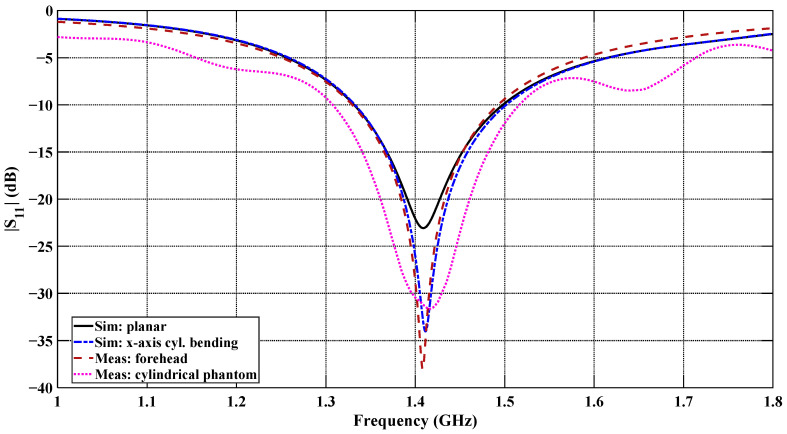
Comparison of measured and simulated |*S*_11_| responses of the designed CCSRR-based flexible antenna.

Figure 21 shows the measured and simulated reflection coefficient (*S*_11_) of the flexible antenna under the planar and x-axis cylindrical bending configurations, along with the measurements performed on the human forehead and cylindrical phantom configurations. Both the simulated and measured |*S*_11_| responses show very good agreement at the resonant frequency with minor discrepancies. At the centered frequency of 1.4 GHz, the measured *S*_11_ reaches approximately −38 dB for the forehead-mounted configuration and approximately −30 dB for the cylindrical phantom configuration, indicating excellent impedance matching in both cases. These two measured responses show very good agreement at the resonance frequency of 1.413 GHz, with only a small difference due to the curvature and tissue loading variations. Overall, the measured results confirm the stable impedance behavior under realistic on-body and controlled cylindrical phantom conditions.

## 4. Discussion and Conclusions

A novel miniaturized and biocompatible 1.4 GHz CCSRR flexible antenna is reported for non-invasive brain temperature sensing on an infant’s head, serving as a sensor for microwave radiometry. It demonstrates excellent impedance match in the 1.4 GHz frequency band for all studied bending scenarios, both in simulations and experimental measurements. A shielding ground plane serves to reject the external EMI, achieving near-field directivity better than 99.94%. The analysis of the averaged power density vs. depth confirms, through reciprocity, the improved capture of thermal noise power in bent scenarios with more than 3 dB improvement compared to the planar configuration. The result suggests sensitivity to thermal radiation emitted from tissues as deep as ~4.5 cm. The feasibility of accurate temperature estimation is supported by quantitative analysis, which indicates that the received thermal noise power (~10−^17^ W) lies within the detectable range of microwave radiometers. However, the ultimate temperature resolution will depend on system-level parameters such as receiver sensitivity, bandwidth, and integration time. Overall, the excellent impedance match, near-field directivity, and sensing depth, along with properties such as conformability, biocompatibility, and light weight, address the critical limitations of the conventional rigid antennas and are expected to enable accurate deep brain temperature measurement by a sensitive microwave radiometer. This advancement addresses a significant gap in bedside infant care by improving both monitoring and therapeutic capabilities. Future studies will focus on measurements with a realistic multi-layer head phantom of an infant’s head equipped with controlled temperature conditions. Depth profiling requires multi-frequency radiometric operation [26,85,86]. An important direction of future study will be the development of a multi-frequency microwave radiometric system based on the reported antenna structure to enable reconstruction of depth-dependent temperature distributions. Future work will also address the development of a rigorous lumped equivalent circuit model of the CCSRR antenna structure, which will allow for rapid antenna re-design for operation at other frequency bands and will aid the expansion of this single-antenna design into a multi-antenna sensing structure.

## Figures and Tables

**Table 1:** Comparison between the proposed flexible antenna and the rigid antennas for deep-body radiometric thermography.

Properties	Flexible Antennas	Rigid Antennas
**Material Composition**	*Kapton*, *PDMS*, conductive inks (e.g., silver nanoparticles)	FR4, ceramic, metallic sheets
**Mechanical Properties**	bend, stretch, and conform to curved anatomical surfaces	do not conform to curved surfaces
**Biocompatibility**	biocompatible materials suitable for wearable and implantable devices	non-biocompatible unless coated with protective layers
**Weight**	lightweight	relatively heavy due to the use of dense substrates
**Durability**	may degrade under extreme mechanical stress	long-term stability for stationary applications
**Electromagnetic** **Performance**	performance may vary with deformation	consistent performance due to fixed shape and stable substrate
**Fabrication**	requires advanced fabrication techniques, such as screen printing and inkjet printing	uses traditional PCB technology
**Skin Contact Quality**	conformal placement reduces air gaps and improves EM coupling with tissue	prone to air gaps on curved surfaces, especially during motion
**EMI Suppression**	thin flexible substrate with back shielding results in superior EMI suppression	thick rigid substrate is susceptible to EMI even with back shielding
**Environmental** **Impact**	biodegradable materials reduce environmental impact	non-biodegradable, contributes to electronic waste
**Comfort**	superior comfort and reduced pressure points; better for long-term monitoring	less comfortable and not tolerant to frequent patient motion

**Table 2:** List of conductive materials used in the flexible antenna fabrication.

Reference	Conductive Material	Conductivity (S/m)
[58]	Copper	5.8 × 10^7^
[59]	Silver nanoparticle ink	1 × 10^7^
[60]	Polypyrrole (PPy)	40–200
[61]	Carbon nanotube (CNT)	Upto 10 × 10^3^
[62]	Polystyrene/silver (PS@Ag) hybrid	4.12 × 10^4^

**Table 3:** List of substrate materials used in the flexible antenna fabrication.

Reference	Substrate Material	Relative Permittivity *ε*_r_	Loss Tangent tan*δ*
[63]	Polyimide (*Kapton*)	3.5	0.007
[64]	Polydimethyl- siloxane (*PDMS*)	2.52–2.82	0.017–0.048
[65]	Liquid Crystal Polymer (LCP)	3	0.0037
[66]	Polyethylene Terephthalate (PET)	3.4	0.03
[66]	Polyethylene Naphthalate (PEN)	3.2	0.045

**Table 4:** Dielectric properties (at 1.413 GHz) and thickness of the four head model tissue layers.

Layers	Thickness (*t*) or Radius (*r*) (mm)	Permittivity *ε*_r_	Conductivity (S/m)
**Scalp**	*t* = 3.5	39.6348	1.0406
**Skull**	*t* = 1.5	12.0425	0.2132
**CSF**	*t* = 1.5	67.7656	2.6700
**Brain**	*r* = 55	47.1727	1.5050

**Table 5:** Antenna resonant characteristics under planar and bending configurations.

Bending Configuration	Lower Cut-Off Frequency *f*_min_ (GHz)	Center Frequency *f*_c_ (GHz)	Upper Cut-Off Frequency *f*_max_ (GHz)	10 dB Impedance Bandwidth (MHz)
**Planar**	1.33	1.412	1.50	170
**Cylindrical** ***x*-axis**	1.33	1.413	1.50	170
**Cylindrical** **y-axis**	1.33	1.412	1.49	160
**Spherical**	1.35	1.425	1.51	160

## Data Availability

The datasets used and/or analyzed during the present study are available from the corresponding author upon reasonable request. All data generated or analyzed during this study are included in this submitted/published article.

## Abbreviations

The following abbreviations are used in this manuscript:

AGNWs	Silver Nanowires
BW	Bandwidth
CSF	Cerebrospinal Fluid
CT	Computer Tomography
CNT	Carbon Nanotube
CST	Computer Simulation Technology
CCSRR	Circular Complementary Split Ring Resonator
EM	Electromagnetic
EMI	Electromagnetic Interference
FET	Finite Element Method
HIE	Hypoxic–Ischemic Encephalopathy
ISM	Industrial, Scientific, and Medical
MIMO	Multiple Input, Multiple Output
MRI	Magnetic Resonance Imaging
MWR	Microwave Radiometry
NFD	Near-Field Directivity
PPy	Polypyrrole
PET	Polyethylene Terephthalate
*PDMS*	Polydimethylsiloxane
RTV	Room-Temperature-Vulcanizing
TBI	Traumatic Brain Injury
